# Blood parameters in growing pigs fed increasing levels of bacterial protein meal

**DOI:** 10.1186/1751-0147-49-33

**Published:** 2007-11-09

**Authors:** Anne Louise F Hellwing, Anne-Helene Tauson, Anders Skrede

**Affiliations:** 1 Department of Basic Animal and Veterinary Sciences, Faculty of Life Sciences, University of Copenhagen, Groennegaardsvej 3, Frederiksberg C, DK-1870, Denmark; 2 Department of Animal and Aquacultural Sciences, Norwegian University of Life Sciences, P.O. Box 5003, Ås, N-1432, Norway; 3 Aquaculture Protein Centre, Centre of Excellence, P.O. Box 5003, Ås, N-1432, Norway

## Abstract

The experiment investigated the effects of increasing dietary levels of bacterial protein meal (BPM) on various blood parameters reflecting protein and fat metabolism, liver function, and purine base metabolism in growing pigs. Sixteen barrows were allocated to four different experimental diets. The control diet was based on soybean meal. In the other three diets soybean meal was replaced with increasing levels of BPM, approximately 17%, 35%, and 50% of the nitrogen being derived from BPM. Blood samples from the jugular vein were taken when the body weights of the pigs were approximately 10 kg, 21 kg, 45 kg, and 77 kg. The blood parameters reflecting fat metabolism and liver function were not affected by diet. Both the plasma albumin and uric acid concentrations tended to decrease (*P *= 0.07 and 0.01, respectively) with increasing dietary BPM content, whereas the plasma glucose concentration tended to increase (*P *= 0.07) with increasing dietary BPM content. It was concluded that up to 50% of the nitrogen could be derived from BPM without affecting metabolic function, as reflected in the measured blood parameters.

## Findings

Bacterial protein meal (BPM) is a new protein source fermented on natural gas, ammonia, and oxygen by *Methylococcus capsulatus *(Bath) (>90%), *Ralstonia *sp., *Brevibacillus agri*, and *Aneurinibacillus *sp. The protein content of BPM is 65–70% and the amino acid composition is comparable to those of fish meal and soybean meal [1]. Rapidly growing bacteria may contain up to 25% nucleic acids on a dry matter basis [2]. The nucleic acid (i.e., ribonucleic acid (RNA) and deoxyribonucleic acid (DNA)) content of BPM is approximately 10%, which is similar to that of yeast [3,4] but much higher than that of soybean meal or fish meal [5,6].

In pig production experiments in which 40–50% of the nitrogen (N) was derived from BPM, slightly improved growth performance in the piglet period was recorded in one experiment [7], whereas another experiment found a reduction in weight gain with increasing BPM level, probably due to suboptimal lysine levels [1]. In growing-finishing pigs, high levels of BPM, replacing soybean meal, could be fed without affecting growth performance [1,7], no clinical health problems related to inclusion of dietary BPM being encountered in any of these studies. Heat production, nitrogen retention, and energy retention were not affected in pigs receiving up to 50% of their dietary N from BPM [8].

Adenine and guanine levels are higher in diets containing BPM than in diets containing fish meal or soybean meal, and the excretion of uric acid has been demonstrated to increase with increasing dietary BPM [9]. Although pigs display uricase activity, and purine bases ought to be completely decomposed to allantoin, this might indicate that the uricase activity is insufficient to metabolize all uric acid to allantoin. This could lead to increased plasma levels of uric acid, and possibly the accumulation of uric acid in joints and kidneys [10].

Investigations in mink, rats and chickens [11-13] have shown that liver cell integrity, purine base metabolism, protein metabolism and fat metabolism might be influenced by dietary BPM. Therefore the aim of the present study was to evaluate whether increasing dietary levels of BPM in pig diets lead to changes in blood parameters reflecting protein and fat metabolism, liver function, and purine base metabolism.

Sixteen barrows were allocated to two blocks (A and B) according to time of birth. Each block contained eight pigs from two litters; one pig from each litter was randomly distributed to one of the four dietary treatments. The control diet (P1) used soybean meal as the main protein source. In the other three diets, soybean meal was replaced with increasing amounts of BPM, and approximately 17% (P2), 35% (P3), and 50% (P4) of the N was derived from BPM in these diets. Pigs were fed once daily. Further details regarding the animals, housing, and diet composition have been presented previously [1,8].

The experimental procedures were approved by Danish national animal-protection legislation and were in accordance with the guidelines approved by the member States of the Council of Europe for the protection of vertebrate animals used for experimental and other scientific purposes [14].

At the times of the four balance and respiration experiments, conducted when the animals had reached live weights of approximately 10, 21, 45, and 77 kg, blood samples were taken from the animals after they had first been fasted overnight. The smallest pigs were placed in a dorsal recumbent position and blood was drawn from the jugular vein. Pigs weighing more than 20 kg were kept standing, the head was held with a nose snare, and samples were drawn from the jugular vein. The blood samples were collected in heparin-coated and ethylenediamine tetraacetic acid (EDTA)-coated vacutainer tubes. The samples were chilled on ice, and the plasma was separated by centrifugation for 20 min at 3000 rpm at 4°C. The plasma samples were frozen at -20°C for later analyses.

Plasma samples in heparin-coated tubes were analysed for uric acid, creatinine, xanthine, and hypoxanthine using high performance liquid chromatography [15]. All other blood analyses were performed on samples taken in EDTA-coated tubes using a Vitros DT II Chemistry System (Johnson and Johnson Clinical Diagnostics, Inc., Rochester, New York, USA). All analyses performed were validated for pig plasma. Freidemanns formula was used for the calculation of VLDL and LDL.

The data were analysed using general linear models (GLM) in SAS [16]. Diet, period, block, and interaction between diet and period were analysed as fixed effects. Values are reported as least square means (LSmeans) and presented with the root mean square error (RMSE) as a measure of variance. Pairwise comparisons were made using the PDIFF option and effects were considered significant if *P *< 0.05. The studentized residuals were plotted against the predicted values. Values deviating more than three standard deviations from normal distribution were carefully investigated. If data were omitted new statistical analyses were run without the outliers and the results from these tests were compared with the first. In none of these cases the deletion of outliers did change the conclusions of the statistical analyses.

Results are presented in Table 1. Total plasma protein concentrations ranged between 5.0 and 5.4 g/dl (*P *= 0.18). There was a tendency for a lower albumin content in P4, where 50% of N was derived from BPM (*P *= 0.07). Albumin is the major plasma protein, and a reduction in albumin may indicate a reduction in protein synthesis. In period 4 the concentration of albumin was 4.4 g/dl, which is above the normal range in pigs of between 1.9 g/dl and 3.9 g/dl [17]. The significantly lower levels of albumin observed in periods 1 and 2 were probably caused by suboptimal levels of dietary lysine [8].

**Table 1:** Effect of increasing dietary content of bacterial protein meal and age on selected plasma parameters in growing pigs.

		Diet^∏^				Period^∏^					*P*-values^‡^	
	*n*^#^	P1	P2	P3	P4	1	2	3	4	RMSE	Diet (D)	Period (P)
Total protein [g/dl]	63	5.2	5.4	5.0	5.1	4.7^C^	5.0^B^	5.2^B^	5.8^A^	0.47	0.18	<0.001
Albumin [g/dl]	63	3.6	3.7	3.5	3.4	3.0^B^	2.9^B^	3.9^A^	4.4^A^	0.31	0.07	<0.001
Urea [mg/dl]	63	11.0	11.1	10.9	11.1	12.7^A^	9.8^B^	9.6^B^	12.0^A^	2.41	1.0	0.001
Ammonia [μmol/l]	63	285.0	288.3	252.6	274.6	300.5^A^	295.7^A^	277.1^AB^	227.2^B^	75.61	0.5	0.04
Alanine aminotransferase [U/l]	59^§^	60.6	61.7	66.6	64.9	42.2^B^	75.8^A^	69.0^A^	66.7^A^	15.59	0.70	<0.001
Aspartate aminotransferase [U/l]	61^¶^	40.2	32.0	34.0	37.5	38.5^AB^	44.1^A^	31.2^B^	29.9^B^	13.87	0.41	0.03
Glucose [mg/dl]	63	103.3	106.8	113.6	111.6	95.0^B^	111.8^A^	117.7^A^	110.7^A^	11.46	0.07	<0.001
Creatine kinase [U/l]	50|	1027	930	749	721	413^C^	771^B^	1032^AB^	1211^A^	387	0.25	<0.001
Cholesterol [mg/dl]	62*	92.2	92.9	97.8	100.3	71.6^B^	100.5^A^	102.0^A^	109.0^A^	15.33	0.41	<0.001
HDLC [mg/dl]	59^&^	35.7	38.9	37.2	41.0	29.4^C^	41.8^A^	35.9^B^	45.8^A^	7.19	0.25	<0.001
LDL [mg/dl]	59^&^	49.5	50.6	53.7	52.0	37.7^B^	51.0^A^	60.2^A^	56.8^A^	12.20	0.81	<0.001
VLDL [mg/dl]	63	7.0	7.6	7.5	7.5	7.5	7.9	7.4	6.8	1.30	0.66	0.11
Cholesterol/HDL	59^&^	2.7	2.6	2.7	2.5	2.7^AB^	2.4^B^	2.9^A^	2.4^B^	0.44	0.64	0.02
Triglycerides [mg/dl]	63	35.7	37.8	37.0	37.6	37.7	39.4	36.8	34.2	6.56	0.82	0.18
Creatinine [μmol/l]	62*	82.3	85.1	85.6	87.5	84.4^B^	73.5^C^	70.4^C^	112.0^A^	9.47	0.50	<0.001
Uric acid [μmol/l]	61^¶^	62.0^ab^	65.8^a^	65.2^a^	55.3^b^	57.5^B^	52.2^B^	68.1^A^	70.4^A^	9.49	0.01	<0.001
Xanthine [μmol/l]	61^¶^	9.5	11.1	9.6	9.3	3.5^B^	5.3^B^	10.7^A^	20.0^A^	5.79	0.83	<0.001
Hypoxanthine [μmol/l]	62*	15.2	16.8	19.4	16.8	22.8^A^	25.5^A^	15.3^B^	4.5^B^	9.66	0.68	<0.001

^# ^One of the pigs was sick during one of the balance periods, so data from this pig were omitted from all datasets.

^§ ^All data for one pig were omitted because of higher ALT levels in all periods; the pig was fed P4.

^¶ ^Two values were characterized as outliers of the dataset and omitted.

| Six samples could not be analysed and seven values were characterized as outliers of the dataset and omitted.

* One value was characterized as an outlier of the dataset and omitted.

^&^Four values were characterized as outliers of the dataset and omitted.

^‡ ^The *P*-values for the interaction between diet and period and the fixed effect of block were non-significant, except for urea (*P *= 0.04 interaction effect between diet and period), uric acid (*P *= 0.01, block effect), and hypoxanthine (*P *= 0.01, block effect).

^∏ ^Diet P1 was the control diet containing no BPM. On diets P2, P3, and P4 approximately 17%, 35%, and 50% of the nitrogen was derived from BPM, respectively. The pigs weighed approximately 10 kg, 21 kg, 45 kg, and 77 kg in periods 1, 2, 3, and 4, respectively.

^a, b ^Values with different superscripts differ significantly, effect of diet (*P *< 0.05).

^A, B, C ^Values with different superscripts differ significantly, effect of period (*P *< 0.05).

Plasma levels of urea and ammonia were similar on all diets. The normal range for urea in plasma is between 10 and 30 mg/dl [17] and some of the measured values were slightly below this range, possibly because samples were taken from fasting animals.

The concentrations of the enzymes alanine aminotransferase (ALT) (*P *= 0.70) and aspartate aminotransferase (AST) (*P *= 0.41) were not significantly affected by diet. One outlier pig on P4 had considerably higher levels of ALT than the other pigs did, and was omitted from the dataset; this did not, however, affect the outcome of the statistical analysis. The other pigs on P4 had normal ALT levels, so it could not be determined whether the single high value was caused by feeding a high level of BPM. In a previous study supplying up to 20% of dietary N from RNA consumption affected neither ALT nor AST concentrations [18]. Supplying 26% of dietary N from another type of bacterial protein meal did, however, cause elevated AST but not ALT concentrations in pigs [19]. Although supplying 50% of dietary N from BPM had no effect on ALT or AST concentrations in our study, it cannot be excluded that higher inclusion levels may affect these concentrations.

The plasma glucose concentration was not significantly affected (*P *= 0.07) by diet, but it increased numerically with increasing dietary BPM; all values were within the normal range [17].

The plasma concentration of creatine kinase tended to decline with increasing BPM level; it did, however, increase with age, reflecting the increasing muscle mass of the animals.

The plasma concentrations of cholesterol, high density lipoprotein (HDL), very low density lipoprotein (VLDL), low density lipoprotein (LDL), triglycerides, and cholesterol/HDL were not affected by diet. Müller et al. [11] have demonstrated a reduction in total cholesterol, LDL, and HDL, but not in VLDL, in mink fed high levels of lipids extracted from BPM. However, the amount of fat from BPM in the pig diets in this experiment was very low compared with the levels used by Müller et al. [11], so the cholesterol-reducing effect of fat from BPM was not expected here.

Xanthine, hypoxanthine, and uric acids are all products of the metabolism of purine bases. The plasma concentration of uric acid decreased in pigs fed the diet with the highest BPM content. This was surprising, because previous studies have demonstrated an increase in urinary uric acid excretion with increasing dietary BPM levels [9], and it was expected that the plasma level would either remain constant or increase. Allantoin was not measured in this experiment, but investigations with other types of bacterial protein and yeast RNA have demonstrated that its level increased in pigs [18-20], suggesting a complete purine base metabolism.

It was concluded that up to 50% of dietary N could be derived from BPM without causing significant changes in the investigated blood parameters, except for the decreasing uric acid levels with increasing BPM content. However, the tendency towards changes in plasma glucose might be an effect of BPM, but further investigations are needed to confirm this.

## Abbreviations

ALT: alanine aminotransferase, AST: aspartate aminotransferase, BPM: bacterial protein meal, DNA: deoxyribonucleic acid, HDL: high density lipoprotein, LDL: low density lipoprotein, N: nitrogen, RNA: ribonucleic acid, VLDL: very low density lipoprotein

## Competing interests

The author(s) declare that they have no competing interests.

## Authors' contributions

ALFH participated in experimental design, carried out the blood sampling and statistical analyses, and drafted the manuscript. AHT participated in the experimental design and in writing the manuscript. AS leads the strategic research programme (see below), and contributed to the experimental design and to writing the manuscript. All authors approved of the final manuscript.
